# CAR-T cells: the long and winding road to solid tumors

**DOI:** 10.1038/s41419-018-0278-6

**Published:** 2018-02-15

**Authors:** Maria Michela D’Aloia, Ilaria Grazia Zizzari, Benedetto Sacchetti, Luca Pierelli, Maurizio Alimandi

**Affiliations:** 1 Department of Clinical and Molecular Medicine, Sapienza University of Rome, Rome, Italy; 2 Department of Experimental Medicine, Sapienza University of Rome, Rome, Italy; 3 Department of Science, University Roma Tre, Rome, Italy

## Abstract

Adoptive cell therapy of solid tumors with reprogrammed T cells can be considered the “next generation” of cancer hallmarks. CAR-T cells fail to be as effective as in liquid tumors for the inability to reach and survive in the microenvironment surrounding the neoplastic foci. The intricate net of cross-interactions occurring between tumor components, stromal and immune cells leads to an ineffective anergic status favoring the evasion from the host’s defenses. Our goal is hereby to trace the road imposed by solid tumors to CAR-T cells, highlighting pitfalls and strategies to be developed and refined to possibly overcome these hurdles.

## Facts

- Unparalleled clinical efficacy has been demonstrated using anti CD19-CAR-T cells to treat refractory B-cell malignancies.
- Many are the challenges imposed by solid tumors for a successful development of CAR T-cell immunotherapy.
- Genetically modified T cells can be alternatively generated using transposons systems (e.g., *Sleeping Beauty*) to stably introduce CARs in T lymphocytes.

## Open questions

- How CARs should be designed and engineered to be effective in the treatment of solid tumors?
- Which strategies need to be developed and refined to improve the balance between favorable and unfavorable effects for better therapeutic benefits?
- What are the priorities for CAR-T cell therapy that must be addressed as they concern safety and efficacy?
- Can the use of genome editing techniques be helpful in generating CAR-armed T lymphocytes suitable for the treatment of solid tumors?

## Introduction

T cells normally build poor or no response against syngeneic transformed cells, (a) for their poor antigenicity, (b) because transformed cells are not phenotypically foreign, and (c) for the generalized immunosuppressive conditions often associated with cancer. For these reasons, adequate immune responses against tumors have seldom been observed, at least in patients treated with chemotherapeutic agents^1,2^. These observations stimulated oncologist and immunologists to boost and activate the T-cell responses against tumor cells, attempts that over the years, never accomplished straightforward clinical results.

Recent knowledge shows that the immune system is kept in shape through a delicate network of signaling pathways delivered by T-cell activating receptors (accelerators) and inhibitory receptors (brakes) to regulate the balance between immune response and immune tolerance^3–5^. This established the platform for developing the “alternative strategy” aimed to take the brakes off the anti-tumor immune responses. The successful use of immune-checkpoint inhibitors in clinical trials highlights the enormous potentials of the immune system to efficiently react against virtually any kind of tumor cell. The advantages in terms of significant antitumor activity, induction of long-lasting responses, and favorable safety profile obtained with the use of checkpoint inhibitors (anti-PD-1/PD-L1 antibodies and anti-CTLA-4), definitely proved the concept that the immune cellular responses may be pivotal to regulate anti-cancer activities^6,7^.

Together with the check inhibition, the adoptive cell therapy (ACT) with chimeric antigen receptor (CAR) redirected T cells is perhaps the most attractive anti-cancer strategy.

CARs encode for transmembrane chimeric molecules with dual function: (a) immune recognition of tumor antigens expressed on the surface of tumor cells; (b) active promotion and propagation of signaling events controlling the activation of the lytic machinery. This system has several advantages: (1) to provide “reprogrammed T-cells” of an *ex-novo* activation mechanism; (2) to brake the tolerance acquired by tumor cells, and (3) bypass restrictions of the HLA-mediated antigen recognition, over-stepping one of the barriers to a more widespread application of cellular immunotherapy^8^.

**Fig. 1:**
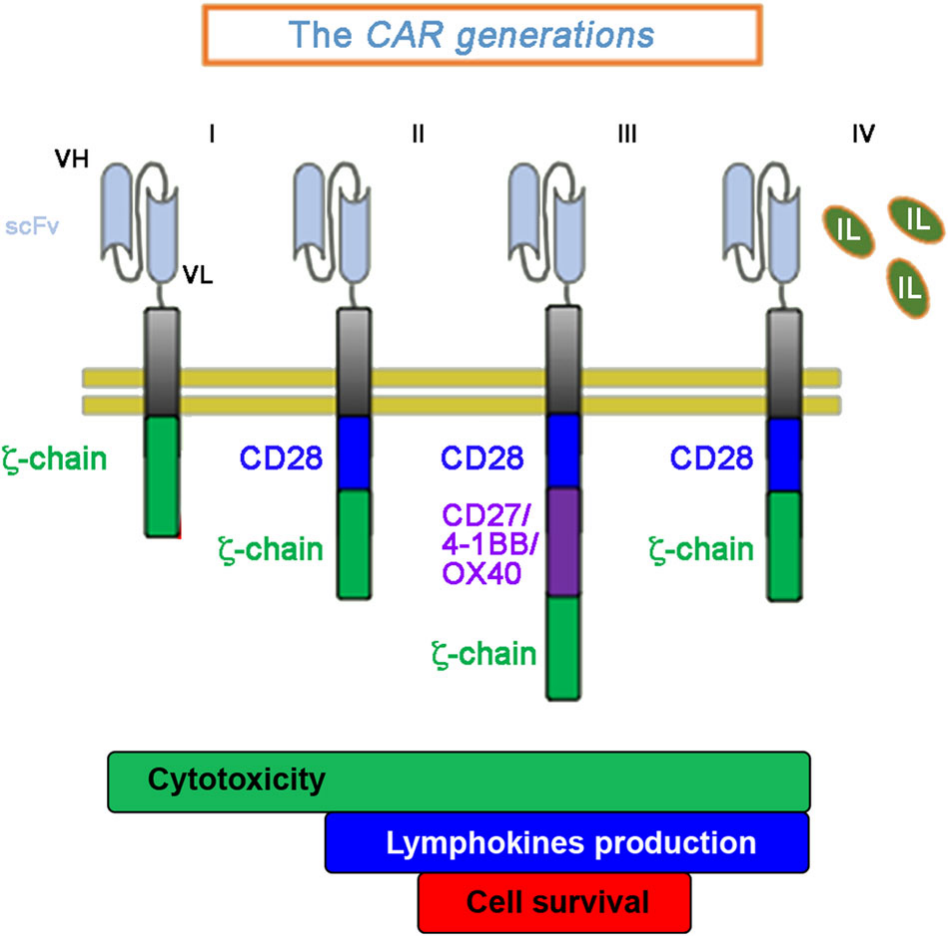
Schematic representation of the chimeric antigen receptors for adoptive cell therapy. CARs comprise an extracellular domain with a tumor-binding moiety, typically a single-chain variable fragment (scFv), followed by a hinge/spacer of varying length and flexibility, a transmembrane (TM) region, and one or more signaling domains associated with the T-cell signaling. The 1^st^ CARs generation is equipped with the stimulatory domain of the ζ-chain; in the 2^nd^ CARs generation the presence of costimulatory domains (CD28) provides additional signals to ensure full activation; in the 3^rd^ generation an additional transducer domain (CD27, 41-BB or OX40) is added to the ζ-chain and CD28 to maximize strength, potency, and duration of the delivered signals; the 4^th^ generation includes armored CARs, engineered to synthetize and deliver interleukins (green ovals)

Eshhar and coworkers were the first to demonstrate that linking the scFv with the TCR ζ-chain or γ-chain for signal transduction, provides T lymphocytes with Ab-type specificity and activates all the functions of an effector cell, including the production of IL-2 and the lysis of target cells^9^. Since then, efforts have been dedicated to produce a number of CARs designed to implement quality, strength and duration of signals delivered by the chimeric molecules. Variability in the functional properties has been obtained by engineering CARs expressing the ζ-chain alone (1^st^ generation) or in tandem with the CD28 (2^nd^ generation), or variably combined with a third signaling domain (3^rd^ generation), such as the 4-1BB (CD137), the OX40 (CD134), ICOS and CD27, with the idea to enhances T-cell proliferation, IL-2 secretion, survival and cytolytic activity. The 4^th^ generation includes “Armored CARs”, designed to increase persistence of engineered T cells in tumor’s microenvironment. Armored CARs combine the CAR functional activities with the secretion of IL-2 or IL-12 expressed as an independent gene in the same CAR vector^10–18^ (Fig.1).

Although the initial attempts to treat patients affected by a variety of solid and liquid tumors, the breakthrough with CAR-T cells therapy was achieved targeting B-cell hematologic tumors.

The use of anti-CD19 CAR T cells have demonstrated consistently high antitumor efficacy in children and adults affected by relapsed B-cell acute lymphoblastic leukemia (B-ALL), chronic lymphocytic leukemia, and B-cell non-Hodgkin lymphoma, with percentage of complete remissions ranging from 70 to 94% in the different trials^19^.

Based on these results, the FDA has approved two immunotherapies with anti-CD19 modified T cells, KYMRIAH [tisagenlecleucel (August 2017)] and YESCARTA [axicabtagene ciloleucel (October 2017)]. These are now a second line treatment for patients up to 25 years of age with B-ALL (KYMRIAH) and for adults with certain types of large B-cell lymphoma (YESCARTA). Similar for the presence of an anti-CD19 murine scFv, they signal through a different costimulatory domain fused in tandem with the CD3 ζ-chain: 4-1BB for KYMRIAH, and CD28 for YESCARTA.

Other B-cell antigens have been targeted in preclinical models, including CD20, CD22, CD23, ROR1, and the kappa light chain. In principle, the treatment of B-cell malignancies with CAR-T cells leads to almost entire B cells repertoire depletion. In this case, the problems derived by the disappearance of B cells from blood can be partially mitigated by immunoglobulins administration. However, depletion of other cell lineages might not be as manageable, and the use of CAR-T cell therapies might be restricted only to specific hematopoietic lineages. In addition, large tumor masses clearance observed in these trials was accompanied by acute and often severe syndrome requiring intensive care, following massive release of cytokines from on-target activated T cells^20–22^^.^

## CAR-T cells therapy for solid tumors

Less exciting conclusions can be derived from clinical trials designed for the treatment of solid tumors with engrafted CAR-T lymphocytes. Although from most of the trials we do not have yet evaluable data, there is enough proof to establish a solid platform for development of CAR-T cells therapy for solid cancers. A good clinical outcome depends on several parameters: (1) choice of the target epitope; (2) CAR architecture; (3) CAR-T cell doses, frequencies and way of administration of CAR-T cells; (4) efficient tumor homing and long-term survival in the tumor environment; (5) patients’ lymphodepletion prior to the administration of CAR-T cells, and subsequent cytokine support.

A key factor responsible for the poor specificity and poor efficacy of CAR-T against malignant epithelial cells is the lack of specific targetable antigens. An ideal target is the signaling active splice variant of EGFR (EGFRvIII), because specifically expressed on glioma cells and indispensable for cell survival^23^. However, encouraging results from early phase trials have been only obtained in neuroblastoma patients treated with anti-GD2 CAR-T cells, and in ErbB2-positive sarcomas treatment^24^. Focus is now on antigens preferentially expressed in certain types of cancers (Table 1).

**Table 1:** Summary of the targetable tumor antigens selected for CAR-T cell-based immunotherapy in epithelial malignancies (from http:/clinicaltrials.gov)

Target antigen	Disease tumor	CAR-T	Gene transfer	Reference	Identifier
CEA	Colorectal carcinoma	CD28/CD3ζ	LV/RV	94, 95	NCT03267173—NCT00673322
	Breast cancer				NCT00673829—NCT02850536
	Liver metastases				
EGFR	Glioma—NSCL cancer	4-1BB/CD3ζ	LV	96	NCT03182816–NCT03152435
					NCT02331693–NCT01869166
EGFRvIII	Glioma—Glioblastoma	CD28/4-1BB/CD3ζ	LV/RV	97, 98	NCT03267173–NCT03170141
					NCT02844062–NCT02664363
					NCT01454596
EphA2	Glioma	NA	NA		NCT02575261
EpCAM	Carcinomas^a^	CD28/CD3ζ	LV		NCT03013712–NCT02729493
					NCT02725125–NCT02915445
ErbB2	Carcinomas^b^	CD28/CD3ζ	RV	24, 47, 99, 100	NCT03267173–NCT02713984
					NCT02547961–NCT01935843
FAP	Mesotelioma	CD28/CD3ζ	RV	101	NCT01722149
FR-α	Ovarian carcinoma	FcεR1 γ	RV	102	NCT03185468–NCT00019136
GD2	Neuroblastoma	CD28/CD3ζ/OX40	RV	103, 104	NCT03356795–NCT02919046
					NCT02765243–NCT02761915
					NCT01822652
GPC3	Lung squamous cell carcinoma	NA	NA		NCT03198546–NCT03084380
	Hepatocellular carcinoma				NCT02905188–NCT02876978
					NCT02723942–NCT02395250
IL13-Rα2	Glioma	4-1BB/CD3ζ	LV	105	NCT02208362
Mesothelin	Metastatic cancer	4-1BB/CD3ζ	LV/RV RNA-EP	106	NCT03356795–NCT03323944
	Pleural mesothelioma				NCT03267173–NCT03182803
	Pancreatic carcinoma				NCT02930993–NCT02792114
	Breast carcinoma				NCT02580747–NCT02465983
	Lung cancer				NCT02388828–NCT01897415
					NCT01583686
MUC1	Carcinomas^c^	CD28/4-1BB/CD3ζ	LV	107	NCT03356808–NCT03356795
					NCT03267173–NCT03198052
					NCT02617134–NCT02587689
MUC16	Ovarian carcinoma	CD28/CD3ζ-IL-12	NA	108	NCT02498912
PSMA	Prostate cancer	CD28/CD3ζ	RV	109	NCT03185468–NCT03089203
					NCT01140373
ROR1	Breast lung carcinoma	NA	NA		NCT02706392
VEGFR-II	Metastatic cancer	NA	RV		NCT01218867
	Melanoma				
	Renal cancer				

*LV* lentiviral, *RV* retroviral, *NA* not available

^a^Gastric cancer—colon carcinoma—hepatocellular carcinoma—pancreatic carcinoma—prostate cancer—esophageal carcinoma

^b^Glioblastoma—glioma—sarcoma—head and neck squamous cell carcinoma—breast cancer—ovarian cancer—gastric cancer—lung cancer—pancreatic carcinoma

^c^Gastric cancer—lung cancer—pancreatic carcinoma—breast cancer—glioma—colorectal carcinoma—hepatocellular carcinoma

Few other factors, besides the differences in the chosen targets, might be responsible for failure of CAR-T cell therapies in solid tumors. The ACT in melanoma requires more cells, more profound lymphodepletion and the use of IL-2 support to obtain optimal results^25–27^. Furthermore, to exploit their cytotoxic function CAR-T lymphocytes need to overcome the limitations imposed by the physical and functional barriers preserving epithelial and mesenchymal compartments. Thus, in perspective, T cells extravasation, tumor homing and persistence in a hostile microenvironment are goals to be accomplished to increase the chances of treating solid tumors with CAR-T cell immunotherapy.

### Extravasation

How to attract CAR-T lymphocytes to solid tumors neoplastic lesions? In principle, activated T cells acquire the expression of a cohort of homing molecules, including E- and P-selectin ligands that mediate T cells rolling on the endothelium, and subsequent chemokines receptors engagement such as CXCR3, CXCL9, and CXCL10. The interaction between chemokines receptors and their ligands facilitates the expression of the LFA-1 and VLA-4 integrins, allowing cell adhesion through to ICAM-1 and VCAM-1, respectively. These features have inspired the engineering of CARs able to target components of the EC matrix, such as αvβ6 integrin^28^ and VEGF receptor-2, usually overexpressed on tumor vessels cells^29^. The idea to target the tumor vascular environment responds to the ability of neoplastic cells to drive angiogenic responses in their favor. EC matrix-directed CAR-T cells would possibly be able to destroy the architecture of the neo-vessels and likely limit the need for T cells to penetrate tumors.

### Inefficient traffic

Trafficking of immune cells toward tumor foci is a dynamic process controlled by a complex network of interactions at multiple levels. The unbalanced secretion of cytokines from tumor cells is one of the major issues responsible for insufficient homing of CD8^+^ CXCR3^high^ T cells at the tumor side. Phenotypically mature T cells express adhesion molecules and chemokines receptors necessary for the interactions with endothelial cells. In particular, the G protein-coupled receptors CXCR3 and CCR5 are often expressed in active tumor-infiltrating lymphocytes (TILs) from melanoma, breast and colorectal cancers, indicating their importance in regulating T-cell trafficking^30^. On the other hand, lack of expression of the cognate ligands, CXCL9 and CXCL10 in many tumor cell types hinders the recruitment of CD8^+^ effector and memory T lymphocytes through chemokines receptors. The tumor endothelium also constitutes a real barrier against T-cell infiltration by overexpressing receptors and ligands. During extravasation, T lymphocytes actively degrade the main components of the sub-endothelial membrane basement and the extracellular matrix, including heparan sulphate proteoglycans (HSPGs)^31^. Therefore, CAR-T cells attacking solid tumors must be able to degrade HSPGs by releasing heparanase (HPSE) to access tumor cells. Recent studies have shown that HPSE deficiency in *in vitro*-engineered and cultured tumor-specific T cells may limit their antitumor activity in stroma-rich solid tumors^32^.

### Tumor microenvironment

To make matters worse, the tumor microenvironment is inhospitable and inaccessible to the invasion of immune cells, because of hypoxia, low nutrients, and for the metabolic acids high concentration that make T cells unable to proliferate and produce cytokines. The absence of important amino acids such as tryptophan, lysine, and arginine, is responsible for the autophagic processes and stress responses activation, inducing T cells to exploit the resources of intracellular nutrients. Immunosuppressive roles have been ascribed to numerous substances produced by tumor and immune cells. Prostaglandin E2 (PGE2) and adenosine are released in large quantities by cancer cells and macrophages in hypoxic conditions, and inhibit T lymphocytes proliferation by activating G protein-coupled receptors and protein kinase A. Moreover, tumor infiltrate is enriched in Tregs, myeloid-derived suppressor cells (MDSC), TAM and TAN (tumor-associated macrophages and tumor-associated neutrophils) favoring tumor survival by the secretion of TGF-β, IL-10, nitric acid, and indoleamine dioxygenase 2–3. CD4^+^/FOXP3^+^ Tregs are suppressor T lymphocytes able to down-modulate immune effector cells activities through multiple mechanisms, including cell–cell contact inhibition and release of soluble factors such as TGF-β and IL-10^33^. To counteract these immunomodulatory activities, CAR-T lymphocytes resistant to TGF-β suppression have been generated by the expression of a dominant negative TGF-β receptor, demonstrating their superior antitumor activity in animal models^34^.

Tumor cells, TILs and immature myeloid cells, are responsible for a large part of ROS production^35^, which can downmodulate CD3-ζ receptor levels, making TCR-mediated T-cell activation less efficient^36,37^. The peculiar aspects of the tumor milieu rich of inflammatory activity provided the rational for constructing CAR-T cells expressing catalase to reduce H_2_O_2_ and counteract the ROS-induced unresponsiveness of these and bystander cells^38^.

IL-4 is another immunosuppressive cytokine that synergizes with IL-10 and TGF-β and promotes activation of macrophages into M2 cells. IL-4’s suppressive effect can be converted into stimulatory effects by chimeric receptors that, engineered to express the IL-4 receptor ectodomain, generate active signals mimicking the IL-2 or IL-7 receptors^39,40^. CAR-T cells expressing “switch” CARs have shown improved capacity to kill TAA-expressing tumor cells^41,42^.

Positive inputs come from experimental use of “Armored CAR” in solid tumor cell therapy. TRUCKS (T cell Redirected Universal Cytokine killing) are engineered to release IL-12 with the intent to mitigate the tumor microenvironment hostile activity. Notably, IL-12 enhances recruitment and functions of innate immunity cells, with the consequence of antigen negative cancer cells increased destruction^42,43^.

Despite Armored CARs have demonstrated superior anti-tumor activity in xenograft models compared to conventional CAR-T, the abundant production of cytokines often results in a severe cytokine release syndrome (CRS) without particular increased efficacy in clinical trials^18^.

## “Safety” and “efficacy”: the two checkpoints of CAR-T cell therapy

The critical point of ACT with chimeric receptors is the need to control unwanted immunological CAR-T cells responses. CAR-T cell therapy’s efficacy is largely counteracted by the occurrence of toxicity, sign at the same time of good performing T-cell activity. For this reason, “safety” and “efficacy” are hallmarks for the improvement of CAR-T cell therapy, and their harmonization will require combination with other therapeutic approaches, to effectively treat solid tumors^44^.

CRS is occurring in almost 80% of the patients treated with anti-CD19 CAR-T cells and can be fatal or life threatening^20,45^. Symptoms include transient neurologic toxicity, febrile neutropenia, cytopenia not resolved by day 28, and infections. This is mostly due to massive release of tumor cell components in the blood for rapid destruction of a large tumor mass (“on-target/on-tumor” toxicity), and to pro-inflammatory cytokines released by CAR-T cells, vascular endothelial cells, and others, resulting in monocyte and macrophage activation with the risk of multiple organ failure.

While CRS can be devastating in course of treatment of liquid tumors, the risk for “on-target/on-tumor” toxicity for solid tumor seems to be lower. This can be at least partially explained by the different stoichiometry in effector-target cell composition reachable in liquid rather than solid tumors, ensuring target killing in a relatively short time. Secondly, the magnitude of CRS correlates with the tumor burden^20,46^.

The other form of toxicity is the on-target/off-tumor toxicity. This is related to the difficulty to identify tumor-specific cell-surface molecules targetable by CAR-T cells without serious side effects. Most antigens are not tumor selective and, particularly in solid tumors, tend to be merely overexpressed. Furthermore, cancer cells redefine over time density and stoichiometry of antigen receptors, with significant implication in predicting the safety profile. For these reasons, the risk of an on-target/off-tumor toxicity is higher in solid tumors and, at least in one case, severity of this reaction may have caused patient death^47^.

In principle, toxicity can be controlled at several levels. One way is to administer required numbers of cells through two (30 + 70)% or three doses (10 + 30 + 60)%, possibly using RNA transiently-engineered CAR-T cells in the first administration to minimize the on-target off-tumor activity^48–50^. Another way is to start the treatment at the earlier stages of tumor development, before the number of cancer cells becomes too high.

**Fig. 2:**
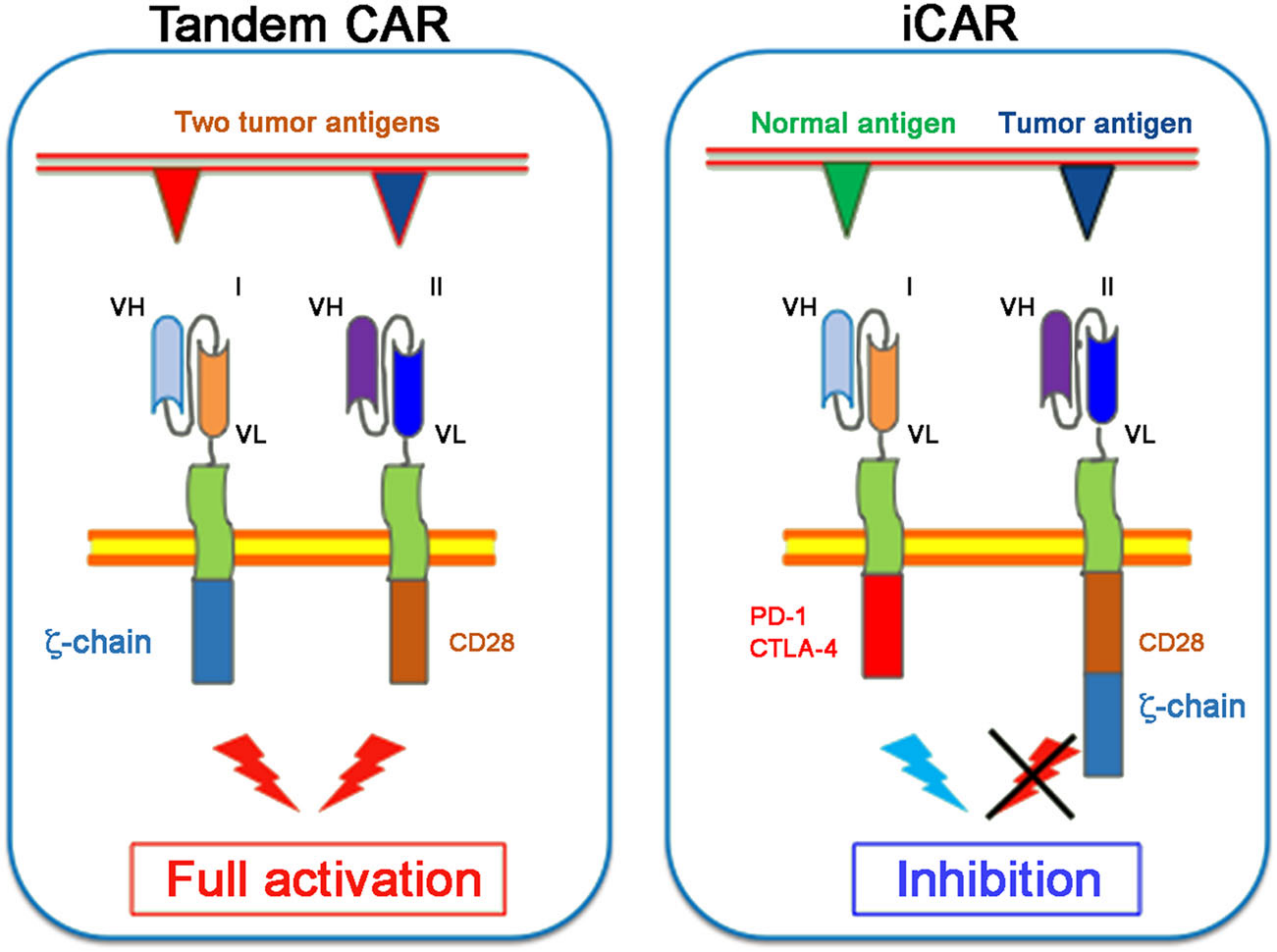
Simultaneous targeting of two antigens may serve to enhances (Tandem CAR) or cut down (iCAR) the activity of the CAR-T cells. Tandem CARs (TanCAR) mediate bispecific activation of T cells through the engagement of two chimeric receptors designed to deliver stimulatory or costimulatory signals in response to an independent engagement of two different tumor associated antigens (TAAs). iCARs use the dual antigen targeting to shout down the activation of an active CAR through the engagement of a second suppressive receptor equipped with inhibitory signaling domains

The need to minimize on- and off-tumor’s reactivity, is the major criterion orienting the conceptual design of dual CAR-T antigen recognition. At least two types of approaches are currently under investigation. In the first approach, target selectivity is ensured by a double recognition of two tumor antigens expressed by the same cell, while the second strategy implies the design of inhibitor chimeric antigen receptors called iCARs, able to divert CAR-T cells activity from normal tissues^51,52^ (Fig. 2).

### Tandem CAR

In the first case, the dual recognition of different epitopes by two CARs diversely designed to either deliver killing (i.e. through ζ-chain) or costimulatory signals (i.e., through CD28) allows a superior activation of the reprogrammed T cells. Consequently, this is a safer path restricting Tandem CAR’s activity to cancer cell expressing simultaneously two antigens rather than one. In this case, the potency of delivered signals in engineered T cells will remain below threshold of activation and thus ineffective in absence of the engagement of costimulatory receptor (CCR)^53^. More importantly, this strategy is potentially suitable to control the on-target/off-tumor toxicity, because the combinatorial antigen recognition enhances selective tumor eradication and protects normal tissues expressing only one antigen from unwanted reactions. In refined strategies for fine-tuning of signals, CARs can be designed to provide weaker strength of activation, while CCR can be equipped with modules for stronger costimulation. The mechanism of dual antigen recognition is also utilized by T lymphocytes in secondary lymphoid organs where T cells receive both activating and costimulatory signals necessary to bust their activity and to sustain their life span during recirculation^54^.

### Inhibitory CAR

Inhibitory CARs (iCARs) are designed to regulate CAR-T cells activity through inhibitory receptors signaling modules activation, typically utilized by T lymphocytes to mitigate the immune responses. This approach combines the activity of two chimeric receptors, one of which generates dominant negative signals limiting the responses of CAR-T cells activated by the activating receptor. iCARs can switch off the response of the counteracting activator CAR when bound to a specific antigen expressed only by normal tissues. In this way, iCARs-T cells can distinguish cancer cells from healthy ones, and reversibly block functionalities of transduced T cells in an antigen-selective fashion (Fig. 2).

In human T lymphocytes, PD-1 and CTLA-4 are inhibitor receptors that reversibly control reduction of TCR signaling potency and can be utilized to mitigate the activation of chimeric receptors. CTLA-4 or PD-1 intracellular domains in iCARs trigger inhibitory signals on T lymphocytes, leading to less cytokine production, less efficient target cell lysis, and altered lymphocyte motility^55^. Critical for the efficacy of iCAR-T cell therapy is antigens selection, because the anti-tumor activity would depend on tissue distribution and stoichiometry between normal and tumor antigens on target cells.

## Gene delivery

An important aspect is the vector’s choice for transferring genes in T lymphocytes. The ideal carrier must meet criteria of efficacy, delivery, and safety. The most used carriers are retroviral (RV) and lentiviral (LV)^56–58^. Both RV and LV systems account for excellent gene transduction efficiency, but differ for the ability to infect resting rather than dividing cells, and for the integration mechanisms that favor privileged areas of the genome rather than others^56–60^.

The ability of LV-based vectors to integrate transgenes in non-replicating cells reduces time of *ex vivo* cell manipulation, with the advantage of obtaining T lymphocytes with a “young” and poorly differentiated phenotype, optimal for therapeutic purposes^61^.

However, the preparation of viral particles for gene therapy remains tedious and carries the risks of contamination by infectious agents, including the replication-competent virus generated by the recombination between vector and packaging cell lines.

The major clinical problems related to the usage of RVs and LVs are the development of innate immune and inflammatory responses to viral vectors^62,63^, and the risk linked to preferential integration near promoters or transcriptional units, with increased chances of causing adverse effects^57–60^.

**Fig. 3:**
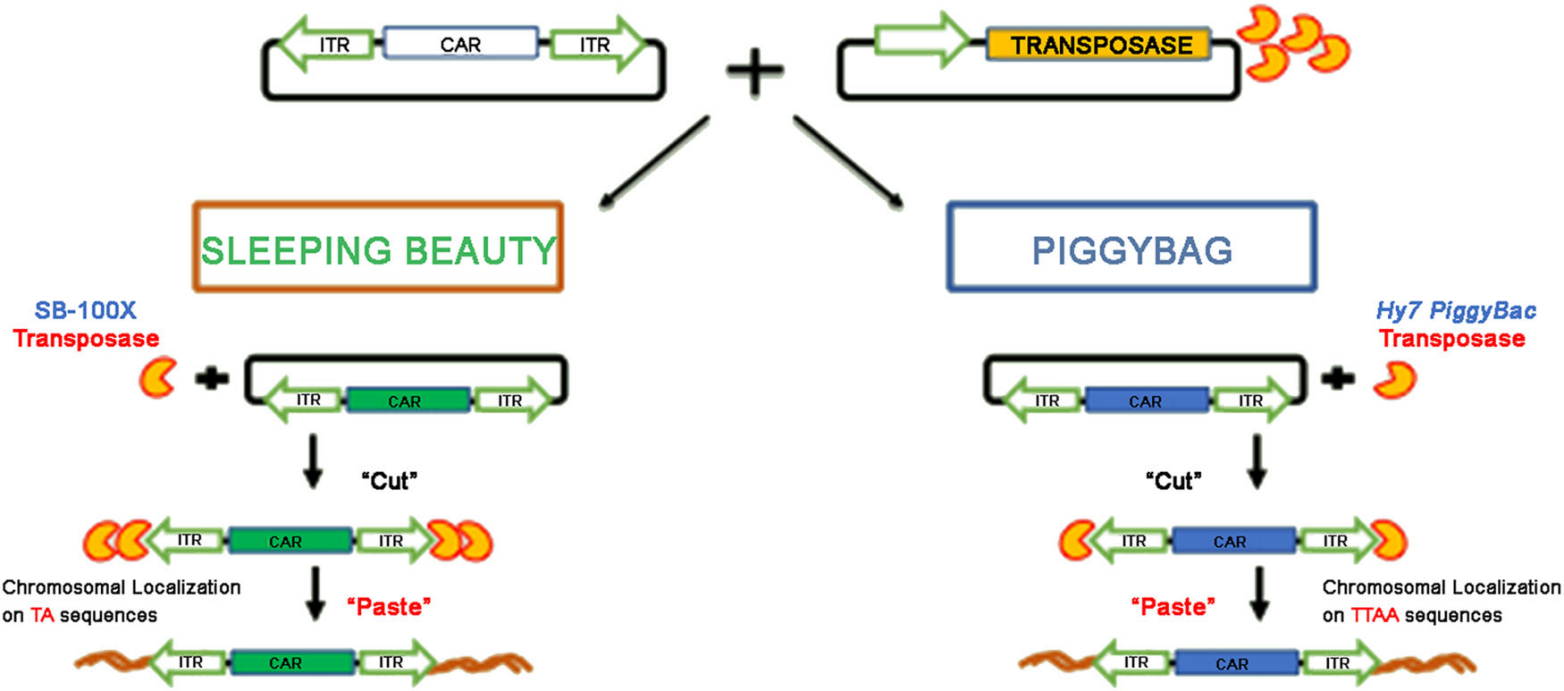
The Transposon systems of *Sleeping Beauty* (SB) and *PiggyBac* (PB) for gene delivery. Transposition is possible through a dual vector system that comprises the transposon containing the transgene flanked by two inverted terminal repeats, and a transposase that mobilizes the transposon. The CAR is integrated into the genome through a cut-and-paste mechanism SB transposon vectors are characterized by the presence of specular IR/DR sequences, target for the transposase. The SB vector contains the gene of interest (CAR). The SB transposase (SB-100×) binds to the IR/DR sequences and cuts the vector to release the transposable portion of DNA. TA sequences in the host DNA act as acceptors of the transposed element. The PB transposon is a mobile genetic element that transposes the gene of interest (CAR) from the vector to the host DNA. The l’*hyperactive PiggyBac* (Hy7 PB) transposase recognizes the transposon-specific “inverted terminal repeats” sequences (ITRs) located at the ends of the gene of interest. Transposition occurs between two TTAA acceptor sites located in the host DNA

**Fig. 4:**
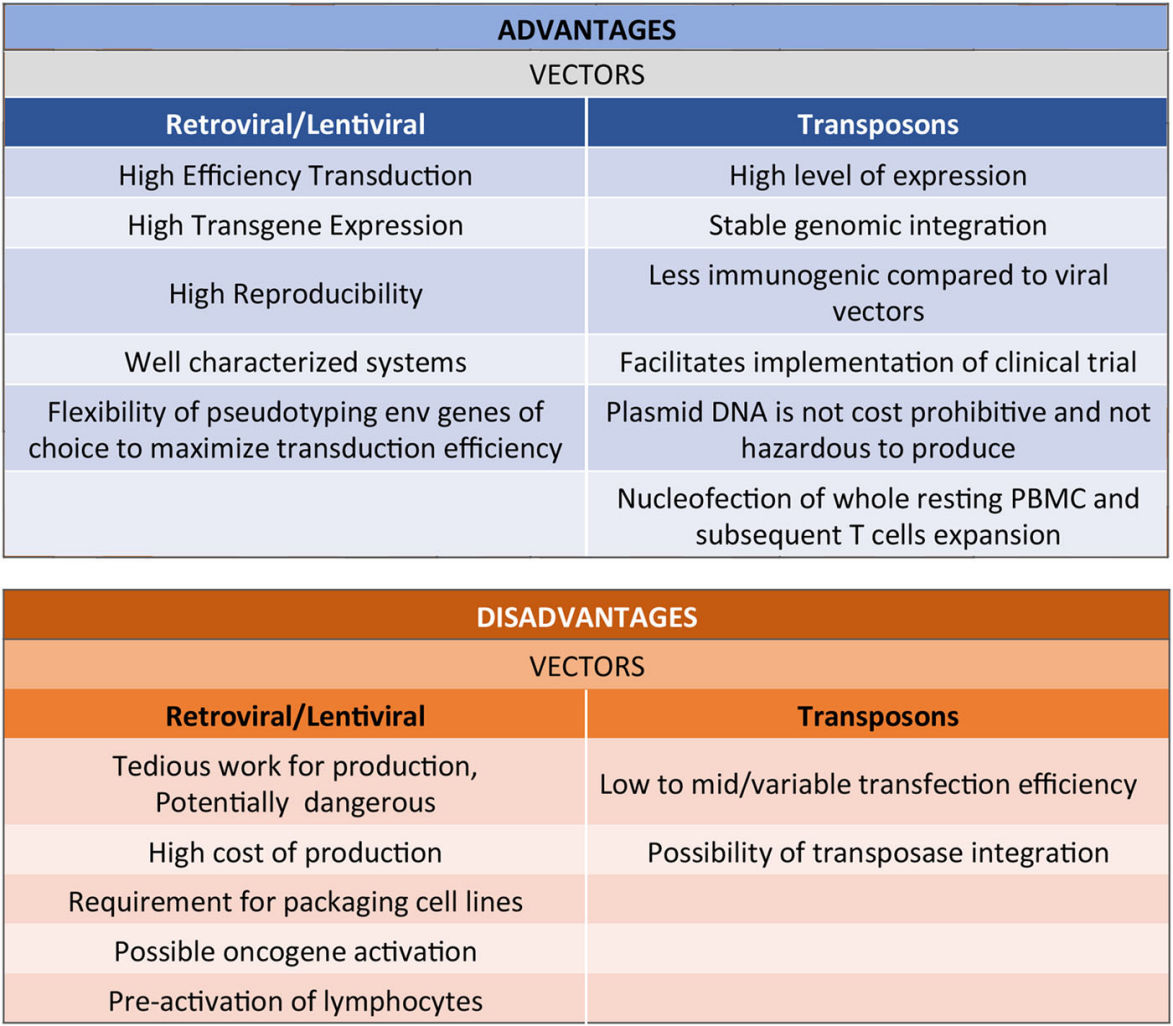
Comparison of viral versus transposon-based gene delivery systems. Plasmid-based transposon systems combine the advantages of integrating viral vectors with those of non-viral delivery systems

Alternative to viral systems are the transposons *PiggyBac* (PB) and *Sleeping Beauty* (SB), allowing integration of large DNA sequences between two ITRs in the host genome by a “cut and paste” transposase’s mechanism^64^ (Fig. 3). The use of transposons in gene therapy would be advantageous for several reasons: simplicity of gene transduction (can be introduced into T cells by nucleofection), safety for both patients and operators, less complexity, minor cost, and less GMP requirements. PB and SB allow excellent standard of gene expression and integration in absence of foreign proteins that can elicit adverse reactions (Fig. 4). Furthermore, since the transposition mechanisms do not involve reverse transcription, transposon vectors are not prone to incorporating mutations and eliminate the risk of rearrangements of the expression cassette that integrates into chromosomal DNA in an intact form^65,66^.

Substantial improvement of these techniques has been demonstrated with the introduction of SB-transposition from minimalistic DNA vectors called Minicircles (MCs). MCs offer more stability, superior gene expression, and less toxicity to T cells compared to SB plasmids^67,68^. The integration profile of a CD19-CAR mobilized through MCs resulted to be highly favorable displaying a near-random integration pattern, in contrast to LVs that prefer for transcriptionally active genes^69^. Several clinical trials in phase I and II are ongoing with SB-generated CD19 CAR-T cells^70^, and one with MUC1-CAR for metastatic breast cancer is currently under review (US-1360).

## The problem of target loss and antigen escape

An emerging threat to CAR-T immunotherapy is the antigen escape that makes CAR-T cells inefficient against cancer cells. CAR-T cells tumor sculpting exerts a selective pressure involving the selection of antigen-negative cells over time. This phenomenon has been described in many clinical studies, including glioblastoma trial with anti-ErbB2-CAR^71^. Appearance of Ag-negative cells limits *per se* the efficacy of immune therapy, highlighting the importance of developing approaches that quickly allow targeting other antigens at the appearance of the new neoplastic phenotype.

Flexibility to the limitation of having one CARs for one antigen has been illustrated in the seminal work of Tamada et al. describing a 3^rd^ generation CAR equipped with an scFv able to bind FITC-labeled monoclonal antibodies directed against tumor antigens^72^. In this case, the anti-FITC CAR-T lymphocytes were able of efficient target lysis, T-cell proliferation, and cytokine production.

**Fig. 5:**
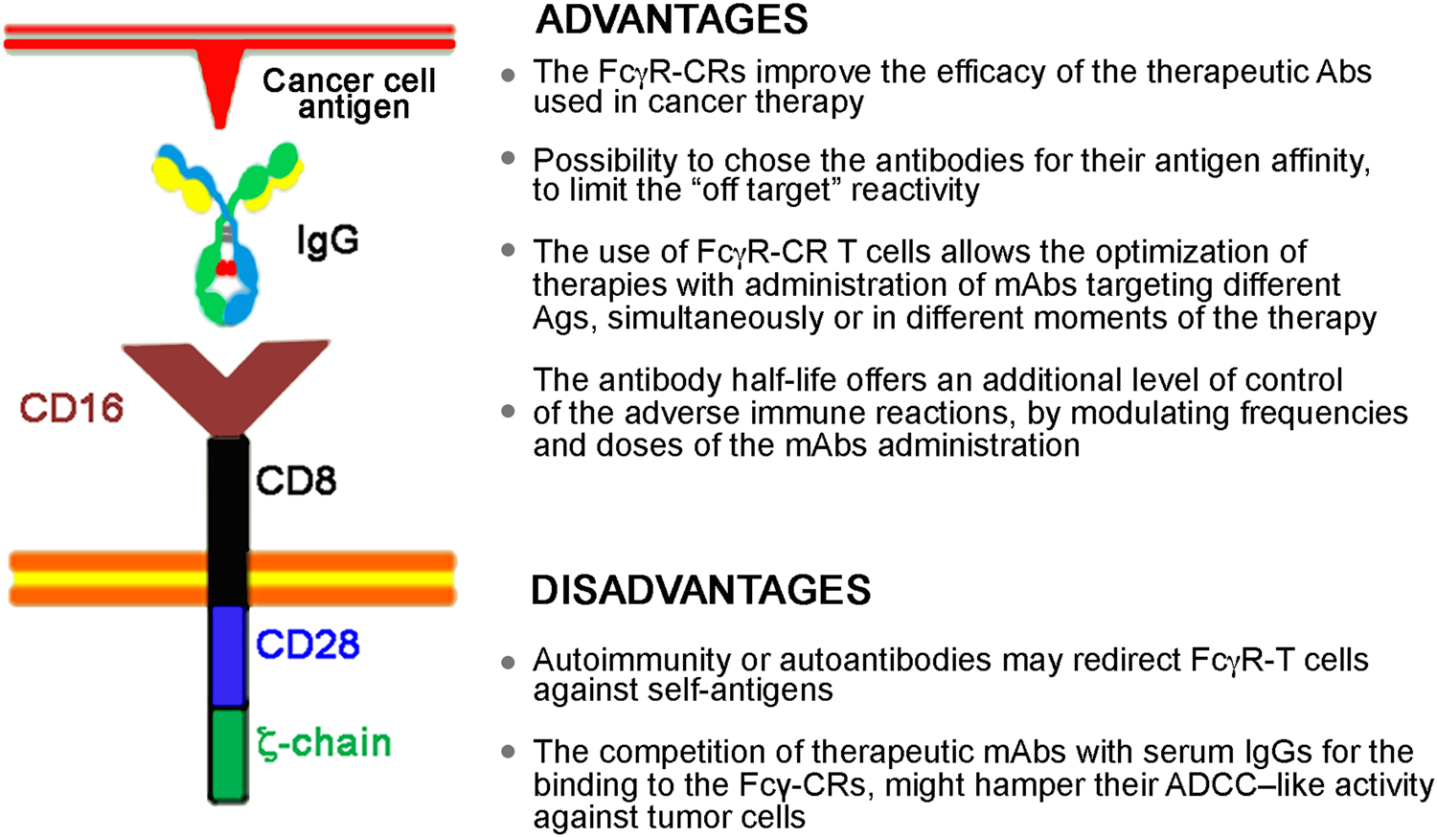
In the CD16-CR, the chimeric receptor extracellular portion is engineered to express an FcγR domain able to complex virtually any mAb directed against TAAs expressed by malignant cells. The FcγR module is combined to a hinge region, a transmembrane domain, and to the intracellular signaling domains of the ζ-chain and CD28. Advantages and disadvantages of the CD16-CR are illustrated

This strategy has been refined by Clemenceau and colleagues by engineering an FcγRIIIa-158(V/V)-FcεRIγ chimeric receptor able to bind any mAb directed against any cancer cell surface antigen^73^. The idea is to combine the therapeutic activity of monoclonal antibodies utilized in cancer therapy with the recruitment of cellular components of the immune system (Fig. 5). The proof of concept has been validated in other laboratories by Kudo K., Ochi F., and D’Aloia MM., engineering CD16-CRs able to complex IgGs with an extracellular FcγRIII binding domain and to deliver biochemical signals through either 4-1BB/ζ-chain or 28-ζ-chain^74–76^. Their FcγRIIIa CRs were able to trigger antibody-dependent cytotoxicity (ADCC)-like activity in transduced T lymphocytes against opsonized CD20^+^ lymphoma cells, *in vitro* or when injected in NOD-*scid-IL2*rg^null^ mice in presence of rituximab.

Further advantage of this strategy is the possibility to control unwanted reactions by the administration of high doses of immunoglobulins that might compete with the FcγR for binding. Moreover, the clearance of the antibodies from blood in about three weeks would offer a further level of control for CD16-CR-T cells activity, protecting patients from GVH reactions. On the other hand, the use of CD16-CR-T cells might be limited by the competition of therapeutic mAbs with serum immunoglobulins for binding to the FcγR-CRs, thus hampering their ability to mediate ADCC. Further restrictions to their usage can be hypothesized for patients affected by autoimmune diseases or diseases mediated by cross-reacting antibodies. In both cases, the high levels of self-reacting Abs might redirect engineered T cells against self-antigens.

## Genome editing

The rapid advancement of genome-editing techniques holds much promise in the field of human gene therapy. By delivering the Cas9 protein and appropriate guide RNAs into cells, the genome can be cut at any desired location, disrupting or changing the sequence of specific genes with the intent to generate armed lymphocytes with increased capabilities of extravasation and survival in tumor microenvironment, or with less potential of toxicity.

Currently, three classes of gene-editing proteins are available: Zinc-Finger, TALENs (Transcription Activator-Like Effector Nucleases), and CRISPR/Cas9 (Clustered Regularly Interspaced Palindromic Repeats/CRISPR-associated-9)^77,78^. Each of them can guide the insertion of the gene of interest in desired sites through the binding of user-defined DNA or RNA sequences, inducing a double-stranded DNA break.

Genome editing has been used to disrupt the TCR complex by targeting either TRAC or TRBC to make T lymphocytes defective of endogenous TCR, less prone to induce off-tumor reactivity in the form of GVHD^78,79^. Also interesting are the knockout of *PD-1*^80^ and *CTLA-4* genes regulating the T-cell checkpoint inhibitors and the silencing of DGK in the TGF-β pathway, made to create the most resistant lymphocytes to immunosuppressive stimuli, including those from tumor microenvironment^81^.

Another possibility is to guide the insertion of transgenes in specific genome sites without affecting endogenous gene structure or expression. Extra-genic regions of the genome called “safe harbors” (GSH) could be able to accommodate the expression of newly integrated DNA without generating adverse effects on the host cell. Three intragenic sites have been proposed as safe harbors (AAVS1, CCR5, and ROSA26) although all of them are in fairly gene-rich regions and are near genes that have been implicated in cancer^82^.

## Concluding remarks

Although majority of CAR T-cell clinical trials are conducted in the setting of hematological malignancies, solid-tumor oncology represents an urgent clinical need. Besides the difficulties of how to reprogram T cells to drive them to tumor sites and survive in the microenvironment, there are few other issues that become more compelling in the perspective of solid tumors CAR-T cells treatment.

One of the major questions would be: which is the best solid tumor to target? The target antigen specificity is a solid selection criterion. As mentioned, the rational for CAR-T cell therapy of glioblastoma is the peculiar expression of the highly specific EGFRvIII antigen in this tumor.

Cancer immunotherapy widely relies on the administration of mAbs directed against signaling receptors or tumor antigens. However, their efficacy and clinical success largely depends on the presence of immune effector cells with ADCC activity in the tumor infiltrate, including NK cells^83–87^. This raises few considerations. It is necessary to identify cancer types where the conspicuous presence of immune cells in the tumor microenvironment would indicate a relative permissive status for engrafted T cells to rich tumor foci and possibly be effective against cancer cells^88-90^. To this extent, several studies have demonstrated that the mutational load and the frequency of neoantigens correlates with the response to immunotherapy in melanoma, lung, and microsatellite instability (MSI)-positive colorectal cancers^91,92^. It is not singular mutations that predict patients’ clinical outcome, but the presence of a high number of mutations and global T-cell responses in the tumor microenvironment. The second is that strategies aimed to combine therapeutic activities of the mAbs used with the potential of a T cell-dependent activation at the tumor site might be ideal. FcγR-CRs can target more than one antigen, sequentially or in combination, thus limiting the risk of immune escape due to emergence or outgrowth of antigen-null tumor cells. However, no clinical trials have been conducted to date to test any of these hypotheses.

Another aspect defining CAR T-cell activity is the affinity for the antigen. High-affinity binding enables CAR driven T-cell effector responses against target cells expressing relatively low levels of antigen^93^. This impacts on a variety of adverse effects occurring immediately or weeks after CAR-T infusion, and there is need as well to invest more research in this field to improve control of off-target toxicity.

There is also the need to implement strategies to control life span of engineered T cells. CAR-T cells can persist and even expand over time, with the consequence of mediating their effects, both therapeutic and deleterious. The introduction of cellular switches to eliminate CAR-T cells in case of adverse events is therefore safe and recommended.

As a final consideration, CAR-T technologies should be validated in preclinical settings using immune competent animals. The immunocompromised mice models cannot recapitulate the immunomodulatory effects of the hosts endogenous immune system, including pathological responses such as CRS, or the immunosuppressive effects generated by the tumor microenvironment on adoptively transferred T cells.

We believe the effectiveness of these living drugs in treating late-stage liquid cancers raised the exciting possibility of a breakthrough approach in cancer therapy, but it needs to be shaped and refined to be impactful in the treatment of solid tumors.
